# Intravascular large B‐cell lymphoma in random deep skin biopsies as a cause of pyrexia of unknown origin

**DOI:** 10.1002/jha2.283

**Published:** 2021-10-03

**Authors:** Kirollos Salah Kamel, May Chiu, Penelope Anne Wright

**Affiliations:** ^1^ Department of Haematology Christchurch Public Hospital Christchurch New Zealand; ^2^ Department of Haematology North Shore Hospital Auckland New Zealand; ^3^ Department of Anatomical Pathology Christchurch Public Hospital Christchurch New Zealand

A 73‐year‐old female presented with a 6 months history of fever of unknown origin, progressive exertional dyspnoea, weight loss, generalised tremors and progressive gait ataxia. Her past medical history was significant for type 2 diabetes mellitus requiring insulin, previous unprovoked pulmonary embolism on rivaroxaban, paroxysmal atrial fibrillation, hypertension and raised body mass index. Extensive investigations had failed to identify any infective, malignant or autoimmune aetiology, and neuroimaging was unremarkable. Importantly, there was no lymphadenopathy or hepatosplenomegaly on cross‐sectional imaging.

As part of her workup, lactate dehydrogenase was measured at 2439 Units/L (reference range 110–220 Units/L) and ferritin was 15,840 μg/L (reference range 20–350 μg/L). A haematology consult was requested and a bone marrow aspirate was done querying haemophagocytic syndrome. No significant haemophagocytosis was identified but immunophenotypic analysis identified a very small population of clonal B cells (0.2% of CD45+ cells), large by forward scatter, positive for CD20 (bright), CD19 (weak), FMC7, CD38 (variable), CD22 (weak) and kappa light chains (moderate to bright intensity) and negative for CD5, CD10, CD11c, CD23, CD138 and CD200. No aberrant T or expanded natural killer cells were detected.

**FIGURE 1:**
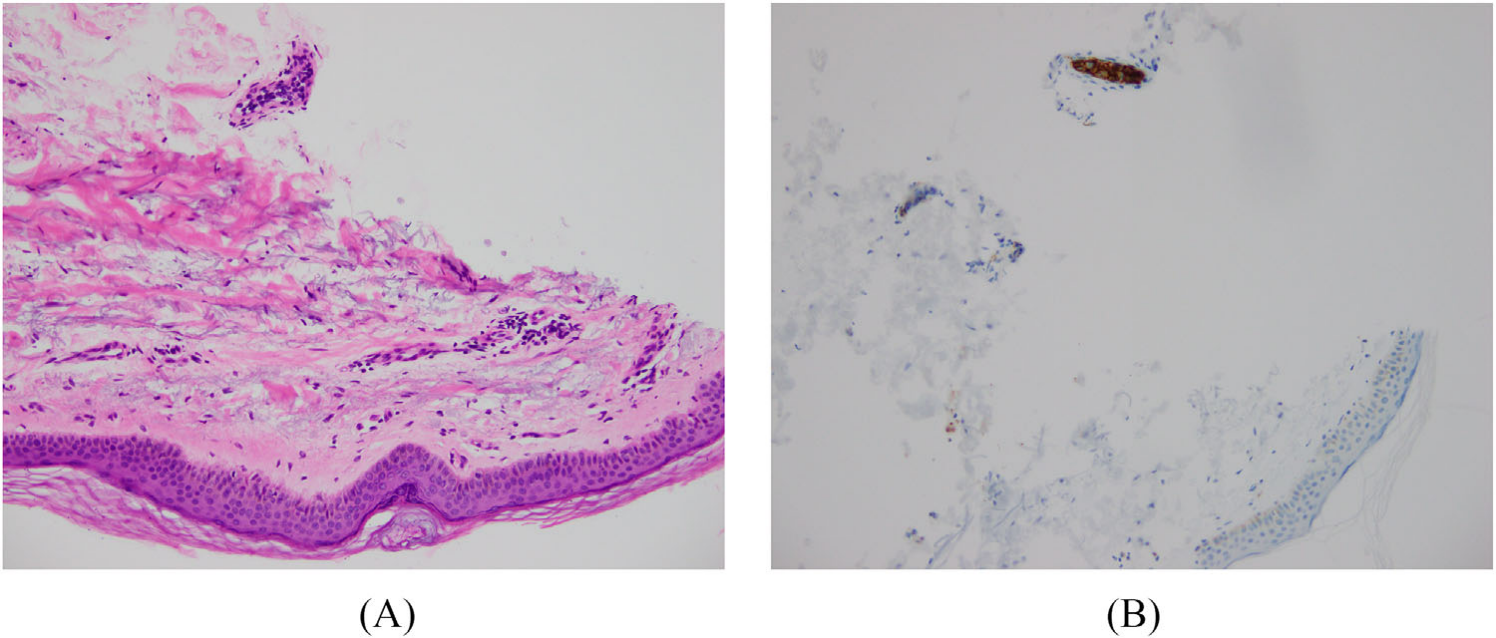
(A) Haematoxylin and eosin stain of a skin punch biopsy including a subcutaneous blood vessel with intravascular large cells. (B) CD20 immunostain demonstrating intravascular large B cells

**FIGURE 2:**
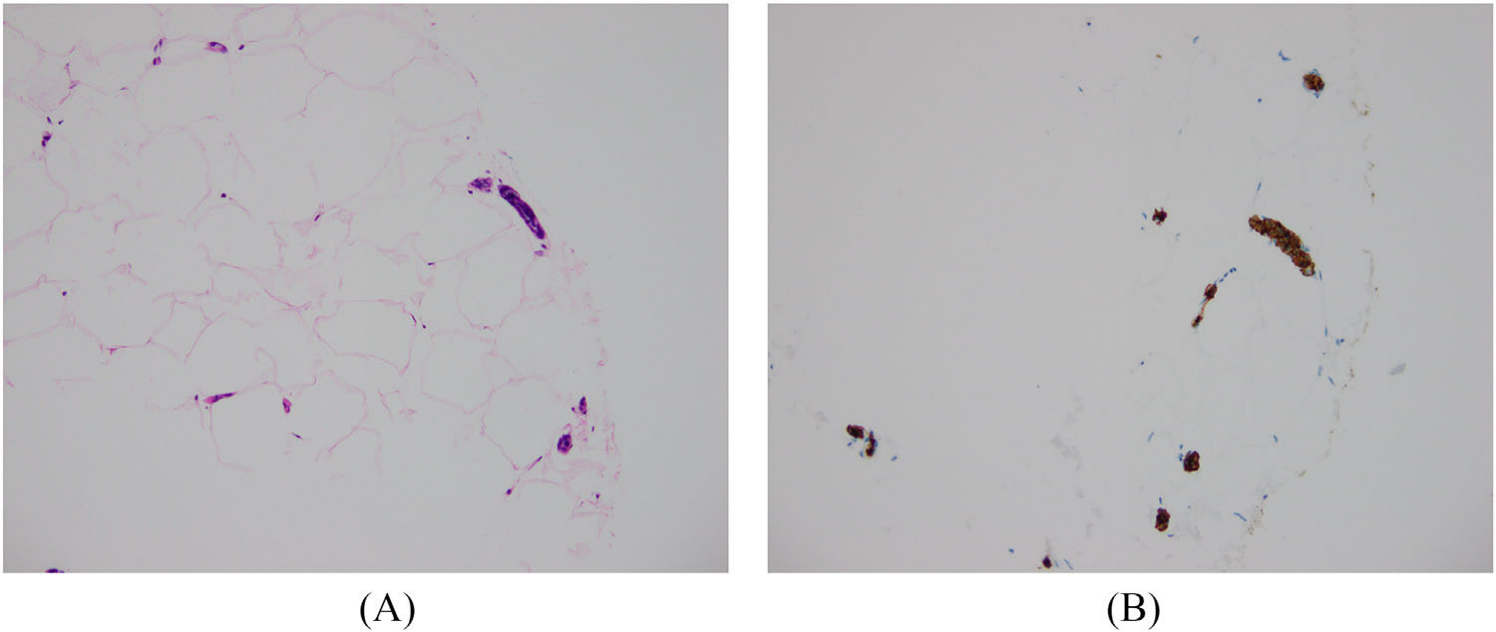
(A) Haematoxylin and eosin stain of subcutaneous adipose tissue in a skin punch biopsy and (B) CD20 immunostain demonstrating intravascular large B cells

**FIGURE 3:**
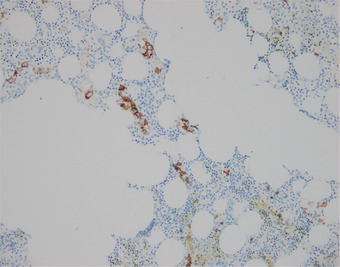
CD20 immunostain on bone marrow trephine showing large B cells in an intrasinusoidal pattern

The possibility of intravascular large B‐cell lymphoma was clinically raised. Random deep skin punch biopsies taken from the left arm demonstrated scattered small vessels within the dermis and subcutaneous adipose tissue containing clusters of large B cells, positive for PAX5, CD20, BCL2, BCL6 and MUM1, and negative for CD10, CD30, cyclin D1 and Epstein–Barr virus encoded RNA in situ hybridisation (Figures 1 and 2). Ki67 was positive in the majority of these cells. A bone marrow trephine biopsy demonstrated intrasinusoidal infiltration with large cells, demonstrated to be B cells by CD20 immunohistochemistry, with Ki‐67 of 80% (Figure 3). Cerebrospinal fluid was negative for involvement with clonal B cells. Cytogenetic studies were not performed. A final diagnosis of intravascular large B‐cell lymphoma was made.

Intravascular large B‐cell lymphoma is a rare type of B‐cell non‐Hodgkin lymphoma that may present with pyrexia of unknown origin without skin lesions. A high index of suspicion is required. Random skin biopsies, in particular incisional biopsies deep enough to sample the subcutaneous tissue, even in the absence of skin involvement are diagnostically useful in the evaluation of patients with suspected intravascular large B‐cell lymphoma. An intrasinusoidal pattern predominates on bone marrow trephine biopsies and can be demonstrated by immunohistochemistry.

